# Mental Health of COVID-19 Patients—A Cross-Sectional Survey in Saudi Arabia

**DOI:** 10.3390/ijerph18094758

**Published:** 2021-04-29

**Authors:** Hasan Saeed Alamri, Wesam F. Mousa, Abdullah Algarni, Shehata F. Megahid, Ali Al Bshabshe, Nada N. Alshehri, Dalal M. Bashah, Roaa Alosaimi, Ahlam Alshehri, Awad Alsamghan, Abdullah Alsabaani

**Affiliations:** 1 Department of Medicine, College of Medicine, King Khalid University, Abha 62527, Saudi Arabia; albshabshe@yahoo.com (A.A.B.); dr_nada@hotmail.co.uk (N.N.A.); 2 College of Medicine, University of Tanta, Tanta 31512, Egypt; wesammousa@hotmail.com; 3 Ministry of Health, Abha 62523, Saudi Arabia; abaid1406@gmail.com; 4 Department of Family and Community Medicine, College of Medicine, King Khalid University, Abha 61421, Saudi Arabia; shehatafarag@yahoo.com (S.F.M.); asoman@kku.edu.sa (A.A.); dr.alsabaani@hotmail.com (A.A.); 5 Biostatistics Department, High Institute of Public Health, Alexandria University, 65 Garidet St., El Horeya Rd., El Shatby, Alexandria 21526, Egypt; 6 King Abdulaziz General Hospital, Jeddah 22421, Saudi Arabia; dr.dalal.08@hotmail.com (D.M.B.); alshehri.ahlam.1410@hotmail.com (A.A.); 7 East Jeddah Hospital, Jeddah 22253, Saudi Arabia; roalosaimi@moh.gov.sa

**Keywords:** mental health, COVID-19, psychological status, anxiety and depression

## Abstract

**Background:** This study aims to investigate the mental health of COVID-19 patients in Saudi Arabia. **Method:** A cross-sectional study was carried out targeting confirmed cases of COVID-19 in Saudi Arabia. Due to travel and time constraints as well as the accessibility of patients, cases were included from East Jeddah Hospital, King Abdulaziz Hospital, and the Oncology Center in Jeddah. The data were collected using a predesigned self-administered questionnaire. The questionnaire addressed COVID-19 cases, personal data, medical history, smoking, traveling abroad, and work-related conditions. Additionally, data regarding contact level with COVID-19 cases were considered. The mental health statuses of the patients were assessed using a validated Arabic version of the Hospital Anxiety and Depression (HAD) scale. The study included 261 COVID-19 patients whose ages ranged from 18 to 65 years. **Results:** The survey findings revealed that 13% of COVID-19 patients had a borderline level of anxiety, 26.8% were considered anxiety cases, while 60.2% were normal. The findings also revealed that 29.9% had a borderline level of depression, 18.4% were considered depression cases, while 51.7% were normal. **Conclusions:** This study concluded that COVID-19 patients experience anxiety and depression, and as the COVID-19 epidemic continues to spread, the results of the study are particularly useful in developing a strategy to psychologically support COVID-19 patients.

## 1. Introduction

Almost 180 countries were affected by the outbreak of Coronavirus Disease 2019 (COVID-19) since its first detection in Wuhan, China, in December 2019 [1,2]. The World Health Organization (WHO) stated that the outbreak created a global public health emergency [3]. According to the WHO [4], the estimated global mortality rate is 3.4%, but the death rates differ between countries and age groups. The COVID-19 pandemic has not only challenged the world’s supply chains and health care systems but also affected the mental health of individuals [5]. The pandemic brought a halt to the hustle and bustle of modern society by imposing lockdowns, which prevented social interactions. The pandemic certainly gave rise to anxiety, fear, and depression with respect to the management of disease and spread of infection [6].

A study conducted by Li [7] investigated the prevalence and predictors of general psychiatric disorders during COVID-19 in the United Kingdom. The study’s findings suggested that people who contracted the virus can experience or develop general psychiatric disorders and tend to feel lonely. The study found that women and young children are considered at high risk of developing general psychiatric disorders and loneliness whereas having jobs and partners were considered protective factors. Another study conducted by Xiang [8] found that people with confirmed or suspected cases of COVID-19 might experience fear of the virus while those in quarantine may experience loneliness, anger, and boredom. Additionally, symptoms of infection such as fever, cough, hypoxia, and body aches as well as the adverse effects of treatment such as insomnia due to corticosteroids can lead to extreme anxiety and mental distress. Wu et al. [9] studied the mental health of the global population, in particular of health care workers, noninfectious chronic disease patients, COVID-19 patients, and quarantined persons. They retrieved 66 studies, including 221,970 participants in their meta-analysis. The overall pooled prevalence of depression, anxiety, distress, and insomnia was 31.4%, 31.9%, 41.1%, and 37.9%, respectively. Noninfectious chronic disease patients, quarantined persons, and COVID-19 patients were at higher risk for depression (*p* < 0.01) and anxiety (*p* < 0.01) than other populations. The general population and nonmedical staff were at lower risk for distress than other populations (*p* < 0.01). Physicians, nurses, and nonmedical staff showed a higher prevalence of insomnia (*p* < 0.01) than other populations.

Other pandemics that have emerged in past years, not only COVID-19, have also left a psychological impact on individuals. A study conducted by Ji [10] found psychological disorders such as obsession–compulsion, hostility, phobic anxiety, paranoid ideation, and anxiety among Ebola survivors. When Severe Acute Respiratory Syndrome (SARS) was first detected, in its early phase, a range of psychiatric morbidities were reported which included continuous depression, panic attacks, anxiety, delirium, and even suicide [8]. It is also worth mentioning that studies performed after the SARS outbreaks showed high rates of anxiety and depression in hospitalized patients [10,11].

Most medical research has been directed at treating fatal respiratory complications, but many patients face psychological obstacles, mainly anxiety and depression [8]. Both of these mental illnesses have considerable impacts on patients infected with this novel, potentially fatal virus. This may be due to fear of the disease itself, loneliness, or worries about family property [7,12]. Apart from fear of contracting the illness itself and from the effects of social isolation on mental health, which lead to anxiety and depression, two other possible factors could add to psychological stress of a disease: the first is when the virus directly affects the central nervous system [13], and the second occurs indirectly, by triggering the immune system and developing “cytokine storms” [14]. In a recent systematic review and meta-analysis that assessed the incidence of depression and anxiety in COVID-19 patients, Leigh-Hunt [15] stated that 45% of COVID-19 patients experienced depression while 47% of patients experienced anxiety. Furthermore, it was suggested that older age and exposure to episodes of low oxygen saturation are additional factors correlated with patients being anxious and depressed, since it was determined that older COVID-19 patients are at increased risk of death and that oxygen saturation is a key indicator in evaluating the severity of the disease [16]. Previous reports showed that females are prone to developing high levels of anxiety and depression [14]. Meanwhile, education level, marital status, having children, pregnancy, smoking, or chronic health conditions may contribute to the mental distress among COVID-19 patients [17] and need to be individually studied. Anxiety and depression are related to lengthier hospitalization and worse outcomes in several diseases [18,19].

Research into depression and anxiety experienced among COVID-19 patients in Saudi Arabia is essential, given that no previous study has been performed in this area. This importance stems from our expectation that the psychological complications of COVID-19 may persist for a long time after recovery. Although we do not have sufficient information about the course of psychological complications as a consequence of infection with COVID-19, studies of previous coronavirus epidemics showed the persistence of psychiatric symptoms and disorders after recovery [20,21]. As an example, a systematic review examined psychiatric disorders in patients hospitalized for severe acute respiratory syndrome (SARS) or Middle East respiratory syndrome (MERS). It assessed the prevalence of mental illness 3 to 46 months after recovery from infection (six studies, *n* > 500 cases) [20]. The point prevalence of anxiety, depressive, and posttraumatic stress disorders were 15%, 15%, and 32%, respectively.

This systematic review also examined psychiatric symptoms in survivors of the 2003 SARS and 2012 MERS epidemics (40 studies, *n* > 1300 hospitalized cases); follow-up occurred 2 months to 12 years after recovery from acute infection [22]. The most common symptom was a frequent recall of traumatic memories, which occurred in 30 percent of patients. Other relatively common symptoms included anxiety; depressed mood; fatigue; irritability; insomnia; and impairments of attention, concentration, and memory. Furthermore, social functioning and role functioning were each impaired among survivors when compared with the general population. Longer-term psychiatric outcomes also included stigma from health care professionals, families, friends, and the general public. The prevalence of long-term psychiatric illness secondary to COVID-19 may be higher than that observed after the SARS and MERS epidemics due to differences in treatments for the viral diseases and the epidemics’ social contexts [23]. For example, the economic crisis caused by the COVID-19 pandemic has surpassed the economic adversity imposed by prior coronavirus epidemics, and the social disruption appears more significant due to its much wider geographical reach. Accordingly, prompt anticipation of mental illness is highly significant in improving outcomes [1]. Therefore, the objective of this study is to determine the mental health statuses of COVID-19 patients if they experience depression and anxiety so that a strategy to support them psychologically can be devised by the health care system. 

## 2. Materials and Methods

### 2.1. Study Design and Sample

A hospital-based cross-sectional study was conducted, targeting COVID-19 patients in Saudi Arabia aged 18 years or more. The treatment policy for COVID-19 cases followed in Saudi Arabia at the time of this study was to hospitalize moderate to severe cases in patients, who have high-risk factors such as obesity, diabetes, or hypertension. As for patients without symptoms or mild symptoms, they were simply instructed to isolate themselves in their homes. Since we did not have the means to contact COVID-19 patients in their homes, only hospitalized patients were recruited for this study. COVID-19 patients admitted to East Jeddah Hospital, King Abdulaziz Hospital, and the Oncology Center in Jeddah from 1 August to 15 September 2020 were included in this study. The researchers collected the data using a predesigned electronic self-administered questionnaire after intensive literature review and expert consultation. A panel of 5 consultants reviewed the questionnaire items independently, and conflict regarding any item was resolved by consensus first and then by voting. The questionnaire inquired about personal data, medical history, smoking, traveling abroad, and work-related conditions. Additionally, data regarding contact level with COVID-19 cases were considered. 

The patient’s mental status was assessed using a validated Arabic version of the Hospital Anxiety and Depression (HAD) scale [24,25]. The Hospital Anxiety and Depression Scale (HADS) was devised 30 years ago by Zigmond and Snaith to measure anxiety and depression in a general medical population of patients. The HAD is short and takes a few minutes to complete while waiting to see the doctor. The HAD uses a 4-point response scale, 0–3, according to the severity of anxiety and depression. The depression domain is measured by seven items, and another seven items measure the anxiety domain. The total score for each domain ranges from 0–21 points. In both domain, cases with scores of 0–7 points are considered normal, cases with 8–10 points are considered to have a borderline abnormality, while scores 11–21 are assessed as cases of anxiety or depression depending on the domain scored. The study included 261 COVID-19 patients whose ages ranged from 18 to 65 years with a mean age of 38.6 ± 10.8 years old. The majority of patients were females (161) and males made up the minority (100).

Before starting the survey, the participants were informed about the study’s aims and personal data protection. They were asked to sign an informed consent form to participate. The study was conducted following the Declaration of Helsinki. It was approved by the King Khalid University Research Ethics Committee (approval number: ECM#2020-237-HAPO-06-B-001) and the Research Ethical Committee at General Directorate of Health Affairs-Aseer Region, Saudi Arabia.

### 2.2. Data Analysis

After the data were extracted, they were revised, coded, and fed to statistical software IBM SPSS version 22 (SPSS Inc., Chicago, IL, USA). All statistical analysis was performed using two-tailed tests. A *p*-value of less than 0.05 was considered statistically significant. Regarding the HAD, all items’ discrete scores were calculated for the depression and anxiety domains separately and categorized with reference to the cutoff points provided above in the Study Design and Sample section. Descriptive analysis based on the frequency and percentage distributions was performed for all variables, including demographic data, work data, and factors associated with risk among COVID-19 cases. Cross tabulation was used to assess the distribution of depression and anxiety statuses according to their personal and work-related data. Relations were tested using the exact probability test due to small frequencies. A line graph was used to assess the association between anxiety and depression scores based on Pearson’s correlation analysis.

## 3. Results

Table 1 demonstrates the study included 261 COVID-19 patients whose ages ranged from 18 to 65 years with a mean age of 38.6 ± 10.8 years old. The majority of patients were females, 161 (61.7%). Regarding educational level, 165 (63.2%) patients were university graduates. As for the work sector, 98 (37.5%) patients were not working, 82 (31.4%) worked in the governmental sector, while 66 (25.3%) worked in the private sector. Regarding income, 120 (46%) cases reported a monthly income of less than 5000 SR and only 16 (6.1%) had an income of 20,000 SR or more. Overall, 167 (64%) patients were married and 57.9% had children.

Table 2 demonstrates the risk factors for COVID-19 infection among study patients. Overall, 72 (27.6%) patients were smokers, 23% had a chronic health condition, and 8 females were pregnant (7.4%). Traveling abroad was reported among 8 cases (3.1%), and 121 patients (46.4%) were in contact with COVID cases. Regarding quarantine days, quarantine exceeded 22 days among 78 (40.8%) cases while 17 (8.9%) cases were quarantined for one week.

Table 3 illustrates anxiety and depression among COVID-19 patients in Saudi Arabia. Exactly 38.7% of the patients felt restless if they have to be on the move, 38.3% of the patients could not sit at ease or feel relaxed, 29.3% of them felt high levels of tension or felt wound up, and 26.4% had worrying thoughts. Among the cases of anxiety, 34 (13%) people had a borderline abnormal level of anxiety while 70 (26.8%) had confirmed cases of anxiety. Exactly 57.4% of the cases could not enjoy the things they used to enjoy, 50.6% could not enjoy a good book, or radio or TV program, 38.3% of the patients lost interest in their appearance, and 36.4% hardly looked forward to enjoying things. Among the cases of depression, 78 (29.9%) people had borderline depression while 48 (18.4%) had confirmed cases of depression.

**Figure 1:**
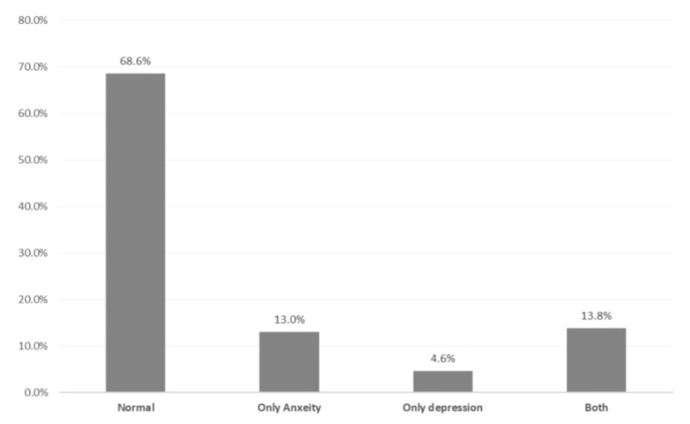
Distribution of study cases according to anxiety and depression statuses.

**Figure 2:**
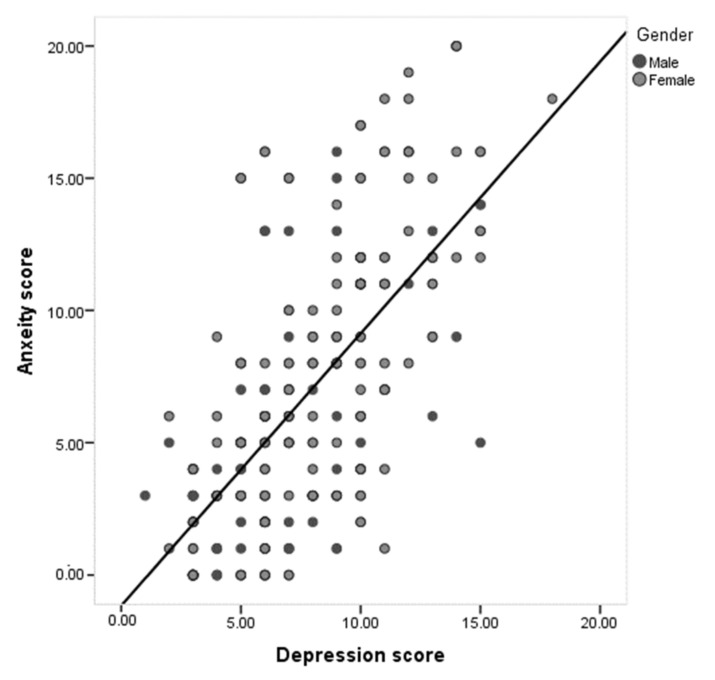
Scatter diagram for correlation between study patients’ depression and anxiety.

Table 4 demonstrates the distribution of COVID-19 cases for anxiety and depression according to their bio-demographic data. Anxiety was significantly higher among females than males (34.8 vs. 14%, respectively; *p* = 0.001). Additionally, 38.8% of cases aged 25–34 years had anxiety compared to 20% of those aged 55+ years (*p* = 0.027). Considering nationality, anxiety was detected among 30% of Saudi cases compared to 9.8% of non-Saudi cases (*p* = 0.007). Exactly 42.2% of patients who were health care workers had anxiety compared to 23.6% of non-health care workers (*p* = 0.010). Additionally, 35.5% of cases with no children had anxiety compared to 21.9% of those who had six children or more (*p* = 0.048). Depression was detected among 42.2% of females compared to 9% of males, with a reported statistical significance (*p* = 0.002). Additionally, 24.3% of patients with low levels of education were depressed compared to 3.4% of patients with secondary school levels of education (*p* = 0.003). Figure 1 demonstrates the Distribution of study cases according to anxiety and depression statuses, about 14% of cases have both depression and anxiety. Regarding the correlation between a patient’s depression and anxiety (Figure 2), it was clear that there is a significant positive intermediate correlation between depression and anxiety scores among males and females (r = 0.60 for males, r = 0.67 for females, and overall r = 0.65). 

## 4. Discussion

This study aimed to investigate the mental health conditions of COVID-19 patients using a cross-sectional survey that evaluates the occurrence of psychological disorders such as depression, anxiety, and stress. The study’s objective was to determine the mental health statuses of COVID-19 patients if they experience depression and anxiety so that a strategy to psychologically support COVID-19 patients can be devised by the health care system. The number of confirmed COVID-19 cases is still steeply rising worldwide. According to the severity of the illness, patients are either hospitalized, admitted in isolated wards, or self-quarantined at home [26]. The study survey revealed that 13% of COVID-19 patients had a borderline level of anxiety, 26.8% had clear cases of anxiety, while 60.2% were normal. 

Regarding depression, while 29.9% had a borderline level of depression, 18.4% had clear cases of depression, and 51.7% were normal. These anxiety and depressive disorders may be related to the deviation in daily life, low levels of understanding of the disease, and fear of death [27,28]. These results highlight the need to pay attention to patients’ mental statuses and to provide support to relieve their anxiety and depression [29]. The study findings also suggest that the levels of anxiety are significantly higher among females than males (34.8% vs. 14%, respectively; *p* = 0.001). This finding is supported by another study that stated that females are prone to developing higher levels of anxiety and depression [14]. This finding raises the question of whether we can assume that COVID-19 plays a role in this differential finding or perhaps there are social reasons for the differences in anxiety and depression between the genders. Further investigations are needed to clarify this issue. 

A recent systematic review and meta-analysis by Leigh-Hunt [15], which assessed the incidence of depression and anxiety in COVID-19 patients, stated that 45% of COVID-19 patients experienced depression while 47% of patients experienced anxiety, which is significantly higher than the results of the survey in this study. This discrepancy may be related to the difference in time at which the data were collected and the differences in nationality with consequent differences in attitudes and religious beliefs, as the majority of the included patients in Leigh-Hunt’s review were from China. As the COVID-19 epidemic continues to spread, our study results help to develop a psychological strategy to support COVID-19 patients. 

## 5. Conclusions

Depression and anxiety are very common among COVID-19 patients in Saudi Arabia. Policymakers in the health care system should apply the findings discussed within this research to implement necessary actions to alleviate the burdens of the COVID-19 pandemic on mental health. It is also essential to consider how the increased need for mental health services will likely continue in the long term, even though new cases and deaths due to COVID-19 will eventually subside. Additionally, health care professionals and females are at high risk and should be given priority when implementing such interventions. Furthermore, a community health care center should be made accessible to people who experience high levels of psychological disorders. 

## 6. Study Limitations

The present study was conducted in Saudi Arabia, and generalizing the results to other areas of the world is not feasible. Additionally, the data were only collected once upon admission; thus, the investigators were unable to follow patients’ psychological changes over time. Moreover, individuals with COVID-19 who had no symptoms or mild disorders (i.e., who did not require hospitalization) were not included in our study. This study cannot determine a causal relationship between anxiety or depression and the sociodemographic variables as this requires a large, multicenter study that is beyond the scope of this research. Therefore, further multicenter studies are required to investigate the previous queries.

## Figures and Tables

**Table 1:** Personal data of the sampled COVID-19 patients in Saudi Arabia.

Personal Data		No	%
Gender	Male	100	38.3%
	Female	161	61.7%
Age in years	<25 years	45	17.2%
	25–34	85	32.6%
	35–44	79	30.3%
	45–54	42	16.1%
	55+	10	3.8%
Nationality	Saudi	220	84.3%
	Non-Saudi	41	15.7%
Educational level	Below secondary	37	14.2%
	Secondary	59	22.6%
	University/more	165	63.2%
Work	Not working	98	37.5%
	Governmental sector	82	31.4%
	Private sector	66	25.3%
	Military sector	15	5.7%
Monthly income	<5000 SR	120	46.0%
	5000–10,000	54	20.7%
	10,000–20,000	71	27.2%
	>20,000 SR	16	6.1%
Marital status	Single	82	31.4%
	Married	167	64.0%
	Divorced/widow	12	4.6%
Children	No children	110	42.1%
	1–2	44	16.9%
	3–5	75	28.7%
	6+	32	12.3%

**Table 2:** Risk factors for COVID-19 infection among study patients, Saudi Arabia.

Risk Factors with COVID-19		No	%
Pregnant	Yes	8	7.4%
	No	100	92.6%
Smoking	Nonsmoker	189	72.4%
	Ex-smoker	34	13.0%
	Current smoker	38	14.6%
Chronic health conditions	None	201	77.0%
	D.M.	19	7.3%
	HTN	23	8.8%
	Allergic/autoimmune diseases	27	10.3%
	Chronic cardiac conditions	6	2.3%
	Psychological conditions	6	2.3%
	Renal conditions	4	1.5%
	Hypothyroidism	3	1.1%
Was abroad within the last three months	Yes	8	3.1%
	No	253	96.9%
Contact with a confirmed COVID-19 case	Yes	121	46.4%
	No	140	53.6%
Days in Hospital	1–7	17	8.9%
	8–14	63	33.0%
	15–21	33	17.3%
	22+	78	40.8%

**Table 3:** Anxiety and depression among COVID-19 patients, Saudi Arabia.

Anxiety Items		No	%
I feel tense or wound up.	Not at all	102	39.1%
	From time to time, occasionally	83	31.8%
	A lot of the time	41	15.7%
	Most of the time	35	13.4%
I get a sort of frightened feeling as if something awful is about to happen	Not at all	126	48.8%
	A little, but it does not worry me	84	32.6%
	Yes, but not too badly	32	12.4%
	Very definitely and quite badly	16	6.2%
Worrying thoughts go through my mind	Only occasionally	137	52.5%
	From time to time, but not too often	55	21.1%
	A lot of the time	39	14.9%
	A great deal of the time	30	11.5%
I can sit at ease and feel relaxed	Definitely	90	34.5%
	Usually	71	27.2%
	Not Often	79	30.3%
	Not at all	21	8.0%
I get a sort of frightened feeling like butterflies in the stomach	Not at all	126	48.3%
	Occasionally	89	34.1%
	Quite Often	16	6.1%
	Very Often	30	11.5%
I feel restless if I have to be on the move	Not at all	46	17.6%
	Not very much	114	43.7%
	Quite a lot	45	17.2%
	Very much indeed	56	21.5%
I get sudden feelings of panic	Not at all	136	52.1%
	Not very often	64	24.5%
	Quite often	43	16.5%
	Very often indeed	18	6.9%
Anxiety level	Normal (0–7)	157	60.2%
	Borderline (8–10)	34	13.0%
	Abnormal (11–21)	70	26.8%
**Depression Items**			
I still enjoy the things I used to enjoy	Definitely as much	18	6.9%
	Not quite so much	93	35.6%
	Only a little	34	13.0%
	Hardly at all	116	44.4%
I can laugh and see the funny side of things	As much as I always could	186	71.3%
	Not quite so much now	53	20.3%
	Definitely not so much now	16	6.1%
	Not at all	6	2.3%
I feel cheerful	Most of the time	69	26.4%
	Sometimes	100	38.3%
	Not often	68	26.1%
	Not at all	24	9.2%
I feel as if I am slowed down	Not at all	125	47.9%
	Sometimes	80	30.7%
	Very often	25	9.6%
	Nearly all the time	31	11.9%
I have lost interest in my appearance	I take just as much care as ever	110	42.1%
	I may not take quite as much care	51	19.5%
	I don’t take as much care as I should	88	33.7%
	Definitely	12	4.6%
I look forward with enjoyment to things	As much as I ever did	114	43.7%
	Rather less than I used to	52	19.9%
	Definitely less than I used to	78	29.9%
	Hardly at all	17	6.5%
I can enjoy a good book or radio or T.V. program	Often	92	35.2%
	Sometimes	37	14.2%
	Not often	85	32.6%
	Very seldom	47	18.0%
Depression	Normal	135	51.7%
	Borderline	78	29.9%
	Abnormal	48	18.4%

**Table 4:** Distribution of COVID-19 patients with anxiety and depression according to their bio-demographic data.

Factors		Anxiety		*p*-Value	Depression		*p*-Value
		No	%		No	%	
Gender	Male	14	14.0%	0.001 *	9	9.0%	0.002 *
	Female	56	34.8%		39	24.2%	
Age in years	<25 years	12	26.7%	0.027 *	7	15.6%	0.374
	25–34	33	38.8%		19	22.4%	
	35–44	17	21.5%		15	19.0%	
	45–54	6	14.3%		4	9.5%	
	55+	2	20.0%		3	30.0%	
Nationality	Saudi	66	30.0%	0.007 *	43	19.5%	0.265
	Non-Saudi	4	9.8%		5	12.2%	
Educational level	Below secondary	8	21.6%	0.071	9	24.3%	0.003 *
	Secondary	10	16.9%		2	3.4%	
	University/more	52	31.5%		37	22.4%	
Health care worker	Yes	19	42.2%	0.010 *	12	26.7%	0.115
	No	51	23.6%		36	16.7%	
Marital status	Single	28	34.1%	0.193	16	19.5%	0.342
	Married	39	23.4%		28	16.8%	
	Divorced/widow	3	25.0%		4	33.3%	
Children	No children	39	35.5%	0.048 *	23	20.9%	0.288
	1–2	7	15.9%		11	25.0%	
	3–5	17	22.7%		10	13.3%	
	6+	7	21.9%		4	12.5%	
Pregnant	Yes	4	50.0%	0.190	2	25.0%	0.879
	No	28	28.0%		23	23.0%	
Smoking	Nonsmoker	53	28.0%	0.430	35	18.5%	0.426
	Ex-smoker	6	17.6%		4	11.8%	
	Current smoker	11	28.9%		9	23.7%	
Chronic health conditions	No	56	27.9%	0.487	35	17.4%	0.455
	Yes	14	23.3%		13	21.7%	

*p*: Exact probability test. * *p* < 0.05 (significant).

## Data Availability

The data set used and analyzed during the study is available from the corresponding author upon reasonable request.
